# Massive Automatic Identification System Sensor Trajectory Data-Based Multi-Layer Linkage Network Dynamics of Maritime Transport along 21st-Century Maritime Silk Road

**DOI:** 10.3390/s19194197

**Published:** 2019-09-27

**Authors:** Hongchu Yu, Zhixiang Fang, Feng Lu, Alan T. Murray, Zhiyuan Zhao, Yang Xu, Xiping Yang

**Affiliations:** 1 State Key Laboratory of Information Engineering in Surveying, Mapping and Remote Sensing (LIESMARS), Wuhan University, Wuhan 430079, China; hongshuxifan8140@163.com; 2 State Key Laboratory of Resources and Environmental Information System, Institute of Geographic Sciences and Natural Resources Research, CAS, Beijing 100101, China; luf@lreis.ac.cn; 3 Department of Geography, University of California at Santa Barbara, Santa Barbara, CA 93106, USA; amurray@ucsb.edu; 4 Engineering Research Center for Spatiotemporal Data Smart Acquisition and Application, Ministry of Education of China, Wuhan 430079, China; 5 National & Local Joint Engineering Research Center of Geo-spatial Information Technology, Fuzhou University, Fuzhou 350002, China; zhaozhiyuan@whu.edu.cn; 6 Department of Land Surveying and Geo-Informatics, The Hong Kong Polytechnic University, Hung Hom, Kowloon, Hong Kong, China; yang.ls.xu@polyu.edu.hk; 7 School of Geography and Tourism, Shaanxi Normal University, Xi’an 710119, China; xpyang@snnu.edu.cn

**Keywords:** maritime network, multi-layer dynamics, traffic flow

## Abstract

Automatic Identification System (AIS) data could support ship movement analysis, and maritime network construction and dynamic analysis. This study examines the global maritime network dynamics from multi-layers (bulk, container, and tanker) and multidimensional (e.g., point, link, and network) structure perspectives. A spatial-temporal framework is introduced to construct and analyze the global maritime transportation network dynamics by means of big trajectory data. Transport capacity and stability are exploited to infer spatial-temporal dynamics of system nodes and links. Maritime network structure changes and traffic flow dynamics grouping are then possible to extract. This enables the global maritime network between 2013 and 2016 to be investigated, and the differences between the countries along the 21st-century Maritime Silk Road and other countries, as well as the differences between before and after included by 21st-century Maritime Silk Road to be revealed. Study results indicate that certain countries, such as China, Singapore, Republic of Korea, Australia, and United Arab Emirates, build new corresponding shipping relationships with some ports of countries along the Silk Road and these new linkages carry significant traffic flow. The shipping dynamics exhibit interesting geographical and spatial variations. This study is meaningful to policy formulation, such as cooperation and reorientation among international ports, evaluating the adaptability of a changing traffic flow and navigation environment, and integration of the maritime economy and transportation systems.

## 1. Introduction

The global maritime transportation network is a composite system using ship movement (i.e., bulk, container, and tanker) to serve trade by different complementary and technical means [1,2]. The multi-components (nodes, links, networks, and traffic flows) and multi-layers (bulk, container, and tanker) behave differently according to various transportation modes, such as tramp shipping with demand-based voyages from the origin to the destination market, and liner shipping with regular schedules. Moreover, differences exist in geographic extent regarding marine spaces, such as the geographic scope of different activities, and the social-spatial and socio-cultural dimensions of the marine unit [2,3]. Current geographic conceptualization treats marine space as components linking social and marine systems, useful to explain the complexity of relationships and dynamics [4,5]. Geographic methodologies are therefore important for understanding maritime network dynamics.

Automatic Identification System (AIS) data include an abundance of information both of dynamics navigation (e.g., latitude and longitude coordinates of ships, timestamp record, course over ground, and speed over ground) and static information (e.g., ship flag, Maritime Mobile Service Identity, International Maritime Organization, ship type, ship name, and ship size) for vessels’ spatiotemporal movements [6]. The AIS data could support global maritime network dynamics analysis and modelling, and supplement most of current studies on maritime transportation network, because current research mostly focuses on sample case studies, selected scenarios analysis, sparse empirical data-oriented, and the container network. The 21st-century Maritime Silk Road Initiative (MSRI) is an important concept and plan announced by the People’s Republic of China. It involves more than 60 countries to enhance trade activities, connecting China with Europe and Africa as well as other parts of Asia [7,8,9]. The global AIS data enable maritime network of the countries along the 21st-century Maritime Silk Road (21C-MSR) and other countries to be investigated, and differences between these two groups to be examined, which may provide insights on the possible effects derived from the 21st-century Maritime Silk Road Initiative initiated (MSRI) by China in 2013. However, there are some challenges to figure out the possible impact of MSRI based on the differential dynamics of maritime network of 21C-MSR and other countries, including designing comprehensive manner, collecting enough data to figure out the difference before and after MSRI, and investigating what make them different.

An improved understanding of maritime transportation network changes would aid in evaluating possible and potential effects related to strategy development, and give insights on dynamic trend prediction. This would be a benefit to policy developers and decision makers in designing effective, comprehensive, and adaptive investment strategies, adjusting and optimizing the global maritime transportation and logistics network. This paper proposes a multi-layer spatial-temporal dynamics framework to understand maritime activity. The study presented here is innovative for the following reasons. First, this paper uses massive AIS Sensor trajectory data to construct and analyze maritime shipping network that extends the application of localization and object tracking technology based on sensors systems. Second, this paper extends the timeline method [10] by taking traffic flow into account to characterize maritime network structure. Third, this paper measures global maritime network dynamics by means of disaggregation across several components (nodes, links, network, and traffic flow), and investigates the flow stability. Finally, this paper examines spatially varying impacts by means of interaction dynamics. It also develops lenses for understanding maritime network dynamics. Such results provide reference information for operators in maritime transportation, investors in maritime trade markets, and officers in maritime management.

The remainder of this paper is organized as follows. Section 2 reviews literature on maritime network and time dynamics. The experimental framework used to reveal multi-layer maritime network dynamics is described in Section 3. Section 4 firstly presents the study area and dataset; then, findings are described. Finally, Section 5 presents conclusions.

## 2. Literature Review

Research on maritime network structure mostly focuses on topology using indicators derived from graph theory, such as node degree, shortest path lengths, clustering coefficient, and others, see Table 1. Other indicators can also be used to study network structure, including linkage intensity, linkage tightness, spatial isolation, and linkage concentration [11]. Maritime network topology structure evolution can be further studied through the changes of indicators over time [12,13,14,15,16,17]. The maritime network topology structure studies seek to reveal connectivity, polarization, clustering, robustness, vulnerability, regional inequality, and spatial variation [1,14,18,19,20,21,22].

Most current studies focus on container shipping network, with few considering multi-layer networks [2,23,24,25]. Kaluza et al. [23] revealed that bulk, container, and tanker maritime networks all follow heavy-tailed distribution for connectivity. Ducruet [24] pointed out that coupling of different types of maritime networks through shared common links. Ducruet [2] analyzed the overlap of different types of maritime networks based on linkage analysis, centrality, eccentricity, clustering coefficient, and assortativity coefficient. Peng et al. [25] investigated the vulnerability of the multilayer maritime network through shortest-path and clustering coefficient changes after a cascading-based attack. However, the traffic flow stability of nodes and links and yearly multilayer maritime network dynamics have rarely been studied. This is very useful to reveal the event-related maritime network dynamics. Automatic Identification System (AIS) data could support ship movement pattern extraction and prediction [26], ship behavior analysis [27,28,29], and maritime network construction and analysis [23,30,31]. This paper approaches evaluation of multi-layer maritime network dynamics by means of disaggregation and comparison across nodes, links, network, and traffic flow using global AIS trajectory data. The differences between the maritime network dynamics of 21C-MSR and other countries in 2013 and 2016 are revealed and analyzed. Additionally, the differences in maritime shipping between before and after 21C-MSR included by 21st-century Maritime Silk Road are illustrated. The maritime network dynamics are critical for understanding cross-regional cooperation and maritime trade pattern changes. Such research offers the potential for identifying complementary advantages with joint collaboration and exchanges in maritime shipping and trade, the very goals of the MSRI.

## 3. Methodology

**Figure 1:**
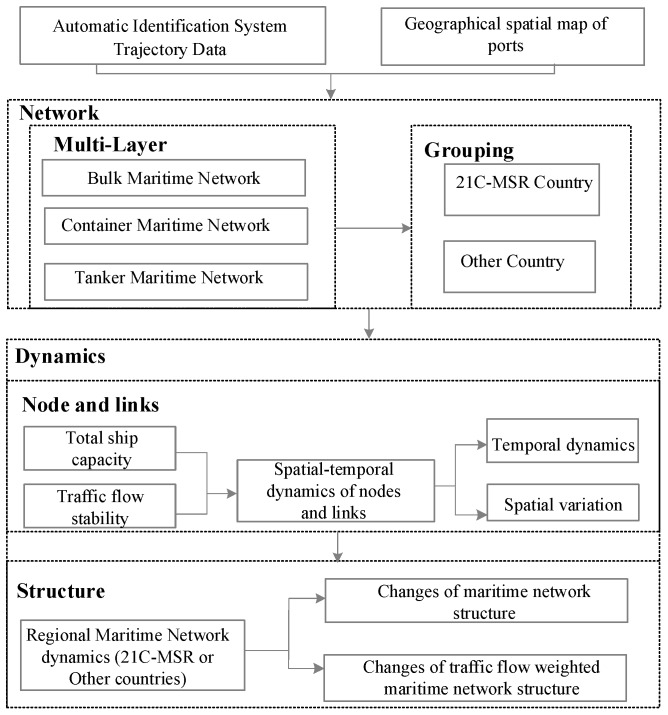
Proposed analytical framework.

Proposed in this paper is an analytical framework for revealing the multi-layer maritime network dynamics of 21C-MSR and other countries, and is illustrated in Figure 1. This framework combines port spatial map and AIS trajectory data to construct a global maritime network based on the origins and destinations of ships using ports. Secondly, the traffic flow characteristics of ports and links can be revealed by means of their shipping capacity and stability. This framework is used to analyze the spatial-temporal dynamics of the maritime network structure and shipping capacity weighted network dynamics for individual countries. Detailed descriptions are provided in the subsections that follow.

### 3.1. Construction of a Maritime Network

**Figure 2:**
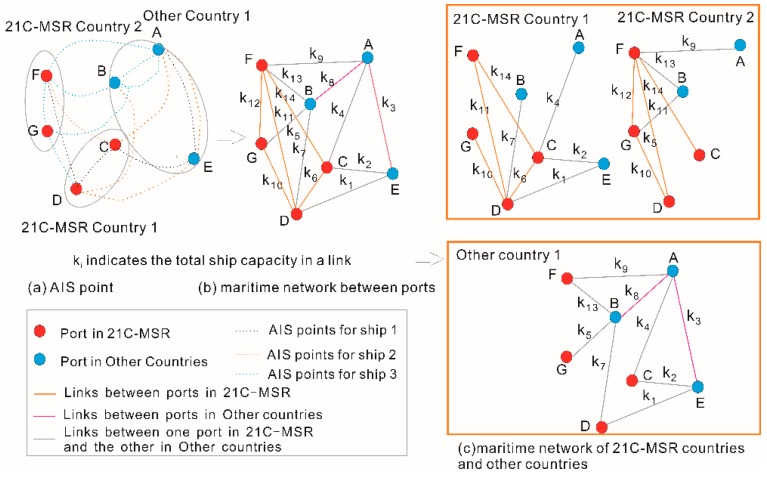
Constructing a maritime network from Automatic Identification System (AIS) data. (**a**) AIS point, (**b**) maritime network between ports, (**c**) maritime network of 21C-MSR countries and other countries.

This section details construction of a time-varying maritime network based on AIS data. Latitude and longitude (location) information for each vessel is known using the AIS data. An example showing ships 1, 2, and 3 is shown in Figure 2a. The time-series locations of these vessels between ports can be viewed as trajectories. Specifically, trajectories exist among ports AE, EC, CD, and DF for ship 1, among ports FC, CA, AE, ED, and DB for ship 2, and among ports AF, FB, BA, AB, BG, GD, DF, and FG for ship 3. Thus, a time-varying maritime network between ports emerges by connecting port pair trajectories as linkages within a pre-specified time unit, such as days, months, seasons, years, or multi-years. Each linkage includes certain properties, such as voyage number and shipping capacity. Figure 2b shows attributes k1, k2, etc. plotted near its corresponding links. The maritime networks for sampled countries, including those of the 21st-century Maritime Silk Road (21C-MSR) and others, are illustrated in Figure 2c. The maritime network of one specific country is based on the criteria that the connections between ports inside the country and between the ports one inside the country and another located in other countries. The connection between the specific country and other countries can be summarized based on the connection between ports one inside the country and another located in other countries.

### 3.2. Maritime Network Dynamics 

The global maritime network can be represented by G=(V,E), where V represents the node set and E represents the link set. This paper proposes a spatial-temporal approach for revealing the multi-component and multi-layer dynamics in the maritime network based on the characteristics of nodes, links, structure, and traffic flow.

#### 3.2.1. Characteristics of Nodes and Links

Transport capacity and stability is very useful for authorities considering strategies for managing maritime traffic, and also for shipping companies to optimize shipping routes. For example, if a port has a highly dynamic transport capacity, this indicates that port authorities need to record and evaluate usage across different time periods in order to improve efficiency. Therefore, this paper uses the transport capacity and stability to characterize dynamics of nodes and links. The following describes transport capacity [22] for node $V_{k}$ or link $L_{k}$ in time $T_{i}$:

(1) $$\left\{ \begin{array}{l} CP_{\left(V_{k}\;or\;L_{k},T_{i}\right)}=\sum_{n=1}^{N(V_{k}\;or\;L_{k},T_{i})}\left(S_{n}^{\left(V_{k}\;or\;L_{k},T_{i}\right)}Q_{n}^{\left(V_{k}\;or\;L_{k},T_{i}\right)}F_{n}^{\left(V_{k}\;or\;L_{k},T_{i}\right)}\right)\\ LogCP_{\left(V_{k}\;or\;L_{k},T_{i}\right)}=Log(CP_{\left(V_{k}\;or\;L_{k},T_{i}\right)})\\ \;=Log(\sum_{n=1}^{N(V_{k}\;or\;L_{k},T_{i})}\left(S_{n}^{\left(V_{k}\;or\;L_{k},T_{i}\right)}Q_{n}^{\left(V_{k}\;or\;L_{k},T_{i}\right)}F_{n}^{\left(V_{k}\;or\;L_{k},T_{i}\right)}\right)) \end{array}\right.$$

where $CP_{\left(V_{k}\;or\;L_{k},T_{i}\right)}$ represents the transport capacity in node $V_{k}$ or link $L_{k}$ in $T_{i}$; $N(V_{k}\;or\;L_{k},T_{i})$ is the total number of services in node $V_{k}$ or link $L_{k}$ in $T_{i}$, $S_{n}^{\left(V_{k}\;or\;L_{k},T_{i}\right)}$, $Q_{n}^{\left(V_{k}\;or\;L_{k},T_{i}\right)}$, and $F_{n}^{\left(V_{k}\;or\;L_{k},T_{i}\right)}$ equal the vessel size, vessel number, and sail frequency in individual service n, respectively; and $LogCP_{\left(V_{k}\;or\;L_{k},T_{i}\right)}$ represents the logarithm of capacity for node $V_{k}$ or link $L_{k}$ in $T_{i}$.

**Figure 3:**
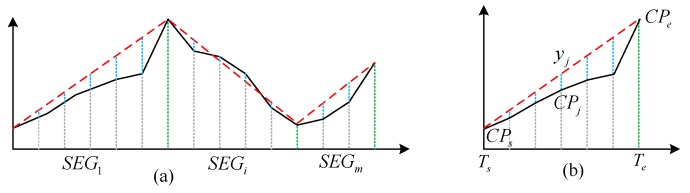
Variation in the capacity of a node or link. The dashed red line indicates the trend in capacity, the black line indicates the real capacity, and the dashed blue line represents the differences. (**a**) Segments division and (**b**) difference calculation for one segment).

Capacity stability for nodes and links can be calculated using the model proposed by [36]. Firstly, the monthly traffic flow curves can be divided into segments using crest or trough, and each segment either monotonically increases, decreases, or remains unchanged. This is shown in Figure 3. The stability of one segment takes into account the trend from the start and end points of an individual segment as well as fluctuation along the segment. This can be characterized as follows:

(2) $$\Delta \bar{F}=\frac{\sum_{j=1}^{p}\Delta F_{j}}{p}=\frac{\sum_{j=1}^{p}\left(CP_{j}-y_{j}\right)}{p}=\frac{\sum_{j=1}^{p}\left\{CP_{j}-\left[\frac{CP_{e}-CP_{s}}{T_{e}-T_{s}}(T-T_{s})+CP_{s}\right]\right\}}{p}$$

(3) $$S_{\left(V_{k}\;or\;L_{k},SEG_{i}\right)}=f_{\left(V_{k}\;or\;L_{k},SEG_{i}\right)}\cdot g_{\left(V_{k}\;or\;L_{k},SEG_{i}\right)}=e^{-|\frac{CP_{e}-CP_{s}}{T_{e}-T_{s}}|}\cdot e^{-\frac{\sqrt{\frac{1}{p}\sum_{j=1}^{p}{\left(\Delta F_{j}-\Delta \bar{F}\right)}^{2}}}{T_{e}-T_{s}}}$$

where $CP_{s}$ and $CP_{e}$ are the capacity at the beginning time $T_{s}$ and ending time $T_{e}$ of the segment, respectively;pis the number of sample points in $SEG_{i}$; $\Delta F_{j}$ is the difference between the real transport capacity $CP_{j}$ and calculated trend value $y_{j}$; and $\Delta \bar{F}$ represents the average value of $\Delta F_{j}$. Equation (2) describes the mean value of the differences between estimation based on the trend and the real capacity, and Equation (3) measures stability calculated by the trend change and the standard deviation of the differences between the estimated and real capacity. Thus, the stability of the link at $T_{i}$ can be derived from the stability of all segments as follows:

(4) $$\begin{array}{l} S_{\left(V_{k}\;or\;L_{k}\right)}=\\ \frac{\sum_{i=1}^{m}S_{\left(V_{k}\;or\;L_{k},SEG_{i}\right)}}{S_{\left(V_{k}\;or\;L_{k},SEG_{1}\right)}+\sum_{i=2}^{m}\left|S_{\left(V_{k}\;or\;L_{k},SEG_{i}\right)}-S_{\left(V_{k}\;or\;L_{k},SEG_{i-1}\right)}\right|+S_{\left(V_{k}\;or\;L_{k},SEG_{m}\right)}+\sum_{i=1}^{m}\Delta T_{\left(V_{k}\;or\;L_{k},SEG_{i}\right)}} \end{array}$$

where $S_{\left(V_{k}\;or\;L_{k}\right)}$ represents the capacity stability of node $V_{k}$ or link $L_{k}$; m represents the segment number of node $V_{k}$ or link $L_{k}$; and $\Delta T_{\left(V_{k}\;or\;L_{k},SEG_{i}\right)}$ represents the duration for the corresponding $SEG_{i}$.

#### 3.2.2. Structure Changes

The timeline method can be employed to evaluate even-related network structure dynamics [10]; thus, this paper compared yearly changes of maritime network structure to reveal the differences between 21C-MSR before and after included by 21st-century Maritime Silk Road, as well as the differences between 21C-MSR and other countries. Consistent with work in this area, considered here is the yearly temporal granularity according to route regularity [37]. The change of one node from time i to i+1 year can be calculated as follows:

(5) $${\tilde{d}}_{\left(T_{i},T_{i+1}\right)}(v)=\left\{ \begin{array}{l} \left|\log\frac{d_{\left(T_{i}\right)}(v)+1}{1}\right|,v\in V(Out\;Node)\\ \left|\log\frac{1}{d_{\left(T_{i+1}\right)}(v)+1}\right|,v\in V(In\;Node)\\ \left|\log\frac{d_{\left(T_{i}\right)}(v)}{d_{\left(T_{i+1}\right)}(v)}\right|+\left|\log\frac{adj_{T_{i}}(v)\cap adj_{T_{i+1}}(v)}{adj_{T_{i}}(v)\cup adj_{T_{i+1}}(v)}\right|,v\in V(Stable\;Node) \end{array}\right.$$

where ${\tilde{d}}_{\left(T_{i},T_{i+1}\right)}(v)$ is the changes in network structure contributed by node v, $d_{\left(T_{i}\right)}(v)$ means the degree of node v at $T_{i}$; $d_{\left(T_{i+1}\right)}(v)$ represents the degree of node v (the count of nodes that have links with node v) at $T_{i+1}$; $adj_{T_{i}}(v)$ represents the neighbors of node v (the collection of nodes that have links with node v) at $T_{i}$; $adj_{T_{i+1}}(v)$ denotes the neighbors of node v at $T_{i+1}$; V(Out Node) represents the collection of nodes in the maritime network at $T_{i}$ but not in the maritime network at $T_{i+1}$, namely as missing nodes; V(In Node) represents the collection of nodes not in the maritime network at $T_{i}$, but rather in the maritime network at $T_{i+1}$, namely as new nodes; and V(Stable Node) represents the collection of nodes both in the maritime network at $T_{i}$ and $T_{i+1}$, namely as stable nodes. The change in network structure can be defined as follows:

(6) $${\tilde{\sigma }}_{\left(T_{i},T_{i+1}\right)}=\left\{ \begin{array}{l} \frac{\sum_{\forall v\in V(OUT)}{\tilde{d}}_{\left(T_{i},T_{i+1}\right)}(v)}{\left|V(g_{T_{i}})\cup V(g_{T_{i+1}})\right|}\\ \frac{\sum_{\forall v\in V(IN)}{\tilde{d}}_{\left(T_{i},T_{i+1}\right)}(v)}{\left|V(g_{T_{i}})\cup V(g_{T_{i+1}})\right|}\\ \frac{\sum_{\forall v\in V(STABLE)}{\tilde{d}}_{\left(T_{i},T_{i+1}\right)}(v)}{\left|V(g_{T_{i}})\cup V(g_{T_{i+1}})\right|} \end{array}\right.$$

where ${\tilde{\sigma }}_{\left(T_{i},T_{i+1}\right)}$ is the maritime network structure changes from $T_{i}$ to $T_{i+1}$ after normalization, including the changes for missing nodes after normalization $\frac{\sum_{\forall v\in V(OUT)}{\tilde{d}}_{\left(T_{i},T_{i+1}\right)}(v)}{\left|V(g_{T_{i}})\cup V(g_{T_{i+1}})\right|}$, for new nodes after normalization $\frac{\sum_{\forall v\in V(IN)}{\tilde{d}}_{\left(T_{i},T_{i+1}\right)}(v)}{\left|V(g_{T_{i}})\cup V(g_{T_{i+1}})\right|}$, and for stable nodes after normalization $\frac{\sum_{\forall v\in V(STABLE)}{\tilde{d}}_{\left(T_{i},T_{i+1}\right)}(v)}{\left|V(g_{T_{i}})\cup V(g_{T_{i+1}})\right|}$ $\left|V(g_{T_{i}})\cup V(g_{T_{i+1}})\right|$ represents the union of the node sets of maritime network at $T_{i}$ and $T_{i+1}$, and can be used to normalize the maritime network structure changes by reducing the differences derived from the network size; $\sum_{\forall v\in V(STABLE)}{\tilde{d}}_{\left(T_{i},T_{i+1}\right)}(v)$ indicates total changes for stable nodes from $T_{i}$ to $T_{i+1}$; $\sum_{\forall v\in V(IN)}{\tilde{d}}_{\left(T_{i},T_{i+1}\right)}(v)$ is the total changes for new nodes from $T_{i}$ to $T_{i+1}$; and $\sum_{\forall v\in V(OUT)}{\tilde{d}}_{\left(T_{i},T_{i+1}\right)}(v)$ is the total changes for missing nodes from $T_{i}$ to $T_{i+1}$.

#### 3.2.3. Weighted Structure Changes

The conventional timeline method can capture the yearly node-link connected structure changes. However, yearly maritime networks may have the same structures, and different transport capacity loaded on nodes and links. Transport capacity changes can reflect the efficiency of maritime transportation, and is very important to maritime policy development. Thus, the weighted structure changes are proposed to analyze both structure and flow changes.

The transport capacity is an important component in maritime network; thus, the transport capacity cannot be ignored. This paper also analyzes the transport flow evolution by means of the proposed capacity weighted timeline method. The transport flow change derived from one node from time i to i+1 can be calculated as follows:

(7) $$\begin{array}{l} {\tilde{d}}_{\left(T_{i},T_{i+1}\right)}{\left(v\right)}^{\prime }=\\ \left\{ \begin{array}{l} \left|\log\frac{\sum_{v_{i}\in E_{\left(T_{i}\right)}(v)}CP_{\left(v,v_{i}\right)}+1}{1}\right|,v\in V(Out\;Node)\\ \left|\log\frac{1}{\sum_{v_{i}\in E_{\left(T_{i+1}\right)}(v)}CP_{\left(v,v_{i}\right)}+1}\right|,v\in V(In\;Node)\\ \left|\log\frac{\sum_{v_{i}\in E_{\left(T_{i}\right)}(v)}CP_{\left(v,v_{i}\right)}}{\sum_{v_{i}\in E_{\left(T_{i+1}\right)}(v)}CP_{\left(v,v_{i}\right)}}\right|+\left|\log\frac{\sum_{v_{i}\in E_{\left(T_{i}\right)}(v)}CP_{\left(v,v_{i}\right)}\cap \sum_{v_{i}\in E_{\left(T_{i+1}\right)}(v)}CP_{\left(v,v_{i}\right)}}{\sum_{v_{i}\in E_{\left(T_{i}\right)}(v)}CP_{\left(v,v_{i}\right)}\cup \sum_{v_{i}\in E_{\left(T_{i+1}\right)}(v)}CP_{\left(v,v_{i}\right)}}\right|,v\in V(Stable\;Node) \end{array}\right. \end{array}$$

where ${\tilde{d}}_{\left(T_{i},T_{i+1}\right)}{\left(v\right)}^{\prime }$ means the change in the transport flow contributed by node v; $\sum_{v_{i}\in E_{\left(T_{i}\right)}(v)}CP_{\left(v,v_{i}\right)}$ represents the capacity of node v at $T_{i}$; $\sum_{v_{i}\in E_{\left(T_{i+1}\right)}(v)}CP_{\left(v,v_{i}\right)}$ represents the capacity of node v at $T_{i+1}$; $\sum_{v_{i}\in E_{\left(T_{i}\right)}(v)}CP_{\left(v,v_{i}\right)}\cap \sum_{v_{i}\in E_{\left(T_{i+1}\right)}(v)}CP_{\left(v,v_{i}\right)}$ indicates the overlap of the capacity of node v between $T_{i}$ and $T_{i+1}$; and $\sum_{v_{i}\in E_{\left(T_{i}\right)}(v)}CP_{\left(v,v_{i}\right)}\cup \sum_{v_{i}\in E_{\left(T_{i+1}\right)}(v)}CP_{\left(v,v_{i}\right)}$ means the integration of the capacity of node v between $T_{i}$ and $T_{i+1}$. V(Out Node) represents the collection of nodes in the maritime network at $T_{i}$, but not in the maritime network at $T_{i+1}$, namely as missing nodes; V(In Node) represents the collection of nodes not in the maritime network at $T_{i}$, but rather in the maritime network at $T_{i+1}$, namely as new nodes; and V(Stable Node) represents the collection of nodes both in the maritime network at $T_{i}$ and $T_{i+1}$, namely as stable nodes. The transport flow changes $\sigma_{\left(t_{i},t_{i+1}\right)}{}^{\prime }$ from $T_{i}$ to $T_{i+1}$ can be calculated as follows:

(8) $${\tilde{\sigma }}_{\left(T_{i},T_{i+1}\right)}{}^{\prime }=\left\{ \begin{array}{l} \frac{\sum_{\forall v\in V(OUT)}{\tilde{d}}_{\left(T_{i},T_{i+1}\right)}{\left(v\right)}^{\prime }}{\left|CP(g_{T_{i}})\cup CP(g_{T_{i+1}})\right|}\\ \frac{\sum_{\forall v\in V(IN)}{\tilde{d}}_{\left(T_{i},T_{i+1}\right)}{\left(v\right)}^{\prime }}{\left|CP(g_{T_{i}})\cup CP(g_{T_{i+1}})\right|}\\ \frac{\sum_{\forall v\in V(STABLE)}{\tilde{d}}_{\left(T_{i},T_{i+1}\right)}{\left(v\right)}^{\prime }}{\left|CP(g_{T_{i}})\cup CP(g_{T_{i+1}})\right|} \end{array}\right.$$

where ${\tilde{\sigma }}_{\left(T_{i},T_{i+1}\right)}{}^{\prime }$ is the transport flow change from $T_{i}$ to $T_{i+1}$ after normalization, including the changes for missing nodes after normalization $\frac{\sum_{\forall v\in V(OUT)}{\tilde{d}}_{\left(T_{i},T_{i+1}\right)}{\left(v\right)}^{\prime }}{\left|CP(g_{T_{i}})\cup CP(g_{T_{i+1}})\right|}$, for new nodes after normalization $\frac{\sum_{\forall v\in V(IN)}{\tilde{d}}_{\left(T_{i},T_{i+1}\right)}{\left(v\right)}^{\prime }}{\left|CP(g_{T_{i}})\cup CP(g_{T_{i+1}})\right|}$, and for stable nodes after normalization $\frac{\sum_{\forall v\in V(STABLE)}{\tilde{d}}_{\left(T_{i},T_{i+1}\right)}{\left(v\right)}^{\prime }}{\left|CP(g_{T_{i}})\cup CP(g_{T_{i+1}})\right|}$; $\left|CP(g_{T_{i}})\cup CP(g_{T_{i+1}})\right|$ represents the integration of capacity between $T_{i}$ and $T_{i+1}$, and can be used to normalize the transport flow changes; ${\sum_{\forall v\in V(STABLE)}{\tilde{d}}_{\left(T_{i},T_{i+1}\right)}(v)}^{\prime }$ represents the total transport flow changes for stable nodes from $T_{i}$ to $T_{i+1}$; ${\sum_{\forall v\in V(IN)}{\tilde{d}}_{\left(T_{i},T_{i+1}\right)}(v)}^{\prime }$ is the total transport flow changes for new nodes from $T_{i}$ to $T_{i+1}$; and ${\sum_{\forall v\in V(OUT)}{\tilde{d}}_{\left(T_{i},T_{i+1}\right)}(v)}^{\prime }$ is the total transport flow changes for missing nodes from $T_{i}$ to $T_{i+1}$.

## 4. Results and Discussion

### 4.1. Study Area and Dataset

AIS data from January 1, 2013 and December 31, 2016 (available at: http://www.myships.com/myships/ and http://www.shipfinder.com/, July 27, 2017) were employed to create an origin–destination (OD) dataset for vessels and connecting ports worldwide. The tanker vessels include transport crude oil, refined oil products, and other chemical oil products. The data categories for each ship are listed in Table 2. All AIS locations for each ship were simplified as a sequence of ports, according to dataset records.

**Figure 4:**
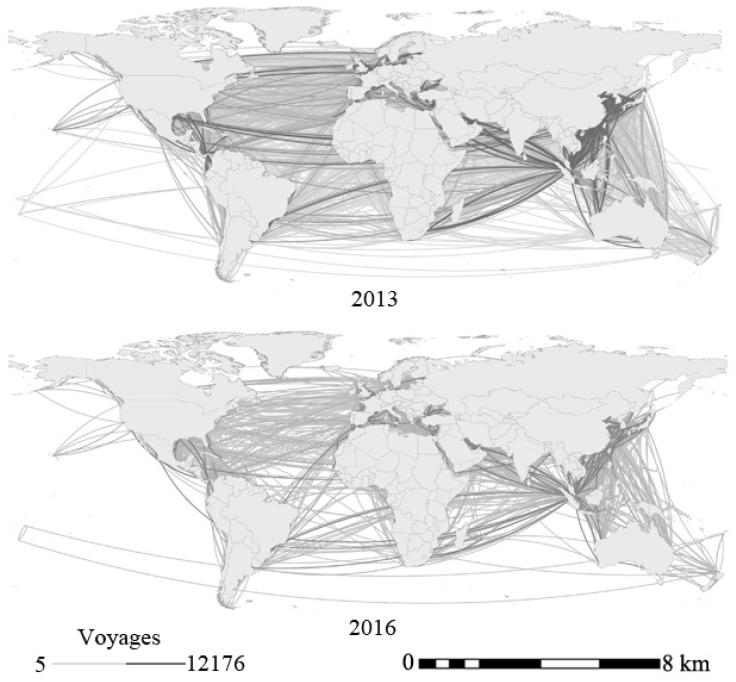
Global maritime network derived from AIS data in 2013 and 2016.

The AIS is compulsory for most commercial ships through the International Convention for Maritime Safety, but loading rates and cargo amount are unavailable [22]. The global maritime network derived from AIS ship data in 2013, 2014, 2015, and 2016 is summarized and can be decomposed by bulk, container, and tanker types to reveal multi-layer dynamics. Figure 4 clearly indicates the different connection patterns and highlight high transport capacity in 2013 and 2016 with the map scale on the bottom right. This paper analyzes the spatial-temporal dynamics of nodes and links, maritime network structures, and traffic flow of 21C-MSR in comparison with those of other countries. 21C-MSR countries include China (CN), Australia (AU), New Zealand (NZ), Republic of Korea (KR), Fiji (FJ), Papua New Guinea (PG), Democratic Republic of Timor-Leste (TL), Brunei (BN), Philippines (PH), Thailand (TH), Indonesia (ID), Cambodia (KH), Malaysia (MY), Laos (LA), Burma (MM), Singapore (SG), Vietnam (VN), Djibouti (DJ), Eritrea (ER), Kenya (KE), Yemen (YE), Oman (OM), United Arab Emirates (AE), Saudi Arabia (SA), Kuwait (KW), Iraq (IQ), Qatar (QA), Iran (IR), Jordan (JO), Turkey (TR), India (IN), Pakistan (PK), Maldives (MV), Sri Lanka (LK), Bangladesh (BD), Morocco (MA), Algeria (DZ), Spain (ES), Italy (IT), France (FR), Greece (GR), Cyprus (CY), Israel (IL), Russia(RU), Romania (RO), and Egypt (EG) [38]. Among these countries, AU, FJ, TL, PH, MA, ES, IT, FR, GR, CY, and SA have been included in 21st-century Maritime Silk Road in 2015, KR, PG, and ER have been included in 2016, and the remaining countries have been included in 2014. Other countries are the remaining countries.

### 4.2. Maritime Network Dynamics of 21C-MSR and Other Countries 

#### 4.2.1. Spatial-temporal Dynamics of Nodes and Links

**Figure 5:**
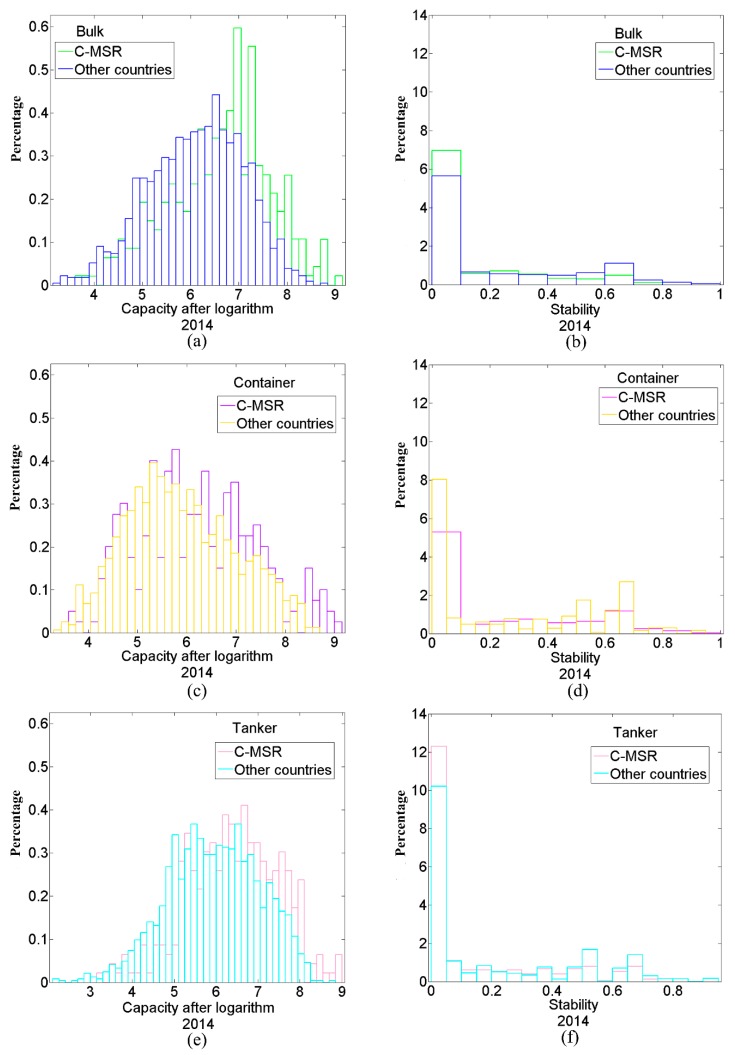
Capacity and stability of ports in 21C-MSR and other countries (sample in 2014). (**a**–**f**) represent bulk capacity after logarithm, bulk flow stability, container capacity after logarithm, container flow stability, tanker capacity after logarithm, and tanker flow stability, respectively.

**Figure 6:**
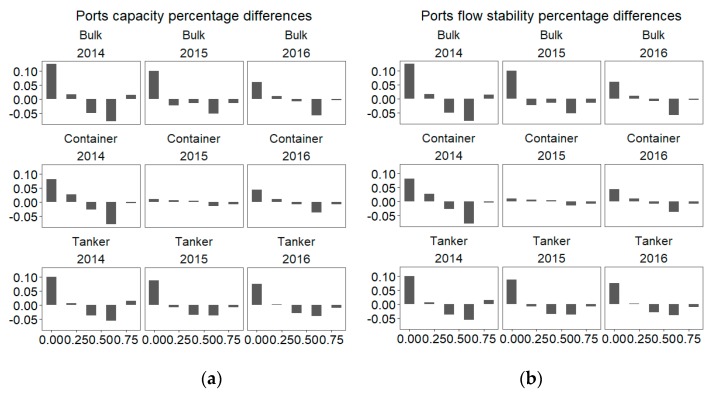
Ports average capacity and stability probability differences between 21C-MSR and other countries. (**a**) Ports capacity percentage differences and (**b**) ports flow stability percentage differences.

The global shipping networks across the study period by bulk, container, and tanker capacity and stability for ports are displayed in Figure 5 and Figure 6. MSRI have been initialed in October, 2013, and corresponding countries continuously have joined the 21C-MSR from 2014. Thus, the contrastive analysis of capacity and stability of ports in 21C-MSR and others countries between 2014 and 2016 have been figured out. Figure 5a,c,e indicate there are more ports that have high bulk, container, and tanker capacity in 21C-MSR than in other countries in 2014. The proportion of ports in 21C-MSR with large bulk, container, and tanker capacity are higher than those in other countries, respectively. There are lower proportions for the ports in 21C-MSR that have low bulk, container, and tanker capacity. The differences between the proportions of ports with high bulk, container, and tanker capacity in 21C-MSR and other countries was narrowed down in 2015 and 2016 compared to 2014 as shown in Figure 6a, which may be related to that newly countries included in 21C-MSR in 2015 and 2016 have a number of ports with lower capacity. Figure 5b,d,f illustrate more ports in 21C-MSR have high changes in bulk, container, and tanker traffic flow in 2014. For example, the proportion of ports with less stable bulk traffic flow in 21C-MSR is higher than that in other countries, but the proportion of ports with more stable bulk flow is lower than that in other countries, the same as container and tanker flow. That indicates some of ports in 21C-MSR present highly dynamics in traffic flow compared to other countries in 2014. Furthermore, the differences between the proportions of ports with high bulk, container, and tanker flow changes in 21C-MSR and other countries was narrowed down in 2015 and 2016 compared to 2014 as shown in Figure 6b, which may be related to that newly countries included in 21C-MSR in 2015 and 2016 have a number of ports with lower flow dynamics.

**Figure 7:**
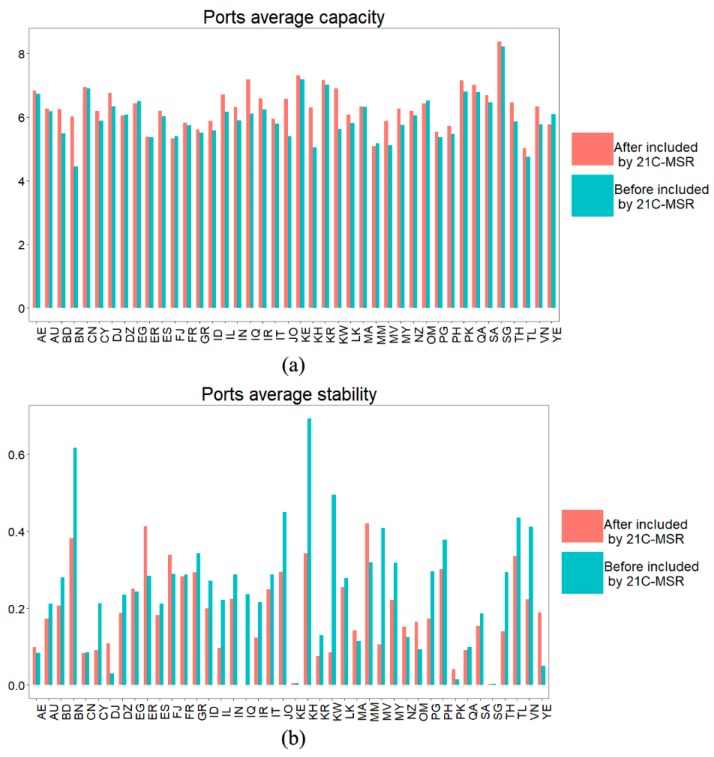
Ports average capacity and stability before and after included by 21C-MSR; (**a**) Port average capacity and (**b**) port average stability.

The ports average capacity and stability in 21C-MSR before and after included by 21st-century Maritime Silk Road are illustrated in Figure 7. Most of the countries have higher average capacity after being included by 21C-MSR than before being included by 21C-MSR. That may be related to more frequent interaction between ports in 21C-MSR after being included by the 21st-century Maritime Silk Road. In more than half of the countries, there exist bigger traffic flow dynamics after being included by 21C-MSR. That indicates that the shipping interaction among ports changes after these countries are included by 21C-MSR.

**Figure 8:**
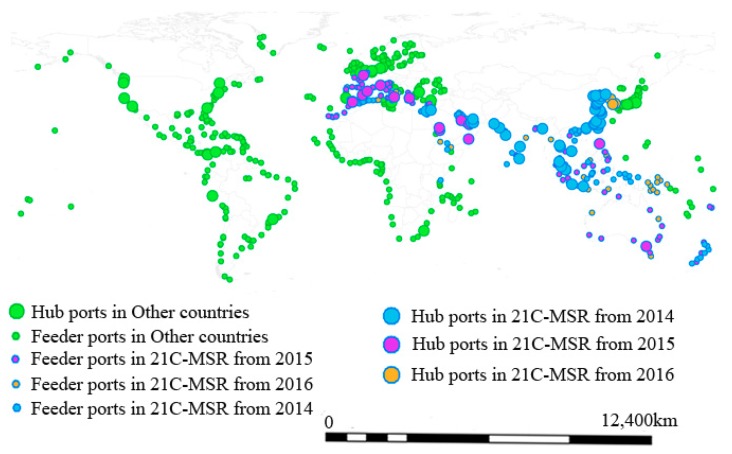
Container ports with low stability in 21C-MSR and other countries.

Figure 8 illustrates container hub and feeder ports with highly flow dynamics between 2014 and 2016 with map scale on the bottom right. The hub ports are the top 100 container ports according to definitive ranking of the world’s largest container ports by Lloyd’s List; the remaining ports are feeder ports. Obviously, there are countries continuously include by 21C-MSR between 2014 and 2016, as well as more ports included by 21C-MSR. In 21C-MSR and other countries, 60.90 % and 50.79%, 57.20% and 55.81%, as well as 62.39 % and 56.62% had container ports with flow stability lower than 0.2 in 2014, 2015, and 2016, respectively. Additionally, there are more hub ports in 21C-MSR than other countries with high flow dynamics. For example, 55 and 32 hub ports with high flow dynamics were located in 21C-MSR and other countries in 2015, respectively. 

Table 3 illustrates that the links of capacity are continuously increasing across the study period in the bulk and tanker layer. There are more links with continuously increasing capacity in bulk maritime network of 21C-MSR than in other countries. The bulk and tanker links with continuously increasing capacity in 21C-MSR represent higher average increased capacity than in other counties. For example, the average increased capacity of bulk and tanker links in 21C-MSR are 3,125,407.29 Dead Weight Tonnage (DWT) and 1,696,798.74 DWT, respectively, whereas in other countries they are 1,243,252.48 DWT, and 1,408,144.76 DWT.

Table 4 illustrates the links of total ships capacity are continuously increasing across the study period in the container layer. There are more links with continuously increasing capacity in container maritime network of 21C-MSR than in other countries, and these container links in 21C-MSR represent higher average increased capacity than in other counties. For example, the average increased total ships capacity of container links in 21C-MSR is 3,524,387.11 DWT, whereas in other countries it is 2,320,540.44 DWT. 38 of 250 links with continuously increasing capacity in other countries are those connected with US and other countries (not considering Origin-Destination in the US), whereas 88 of 388 links with continuously increasing volume in 21C-MSR are those connected with CN and other countries (not considering Origin-Destination in CN). That indicates that CN has more links than the US with continuously increasing container capacity across the study period.

**Figure 9:**
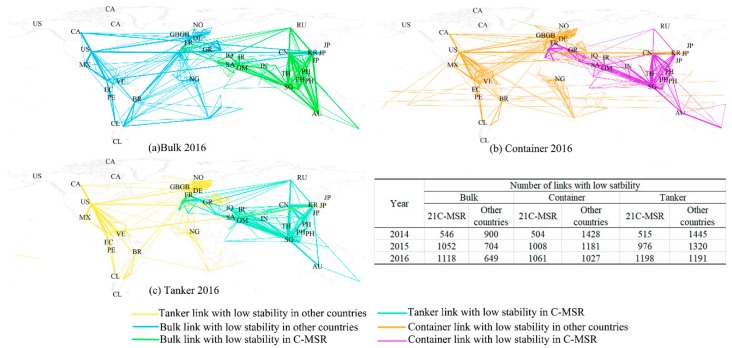
Links with low stability in 21C-MSR and other countries; (**a**) Bulk links with low stability in 2016, (**b**) container links with low stability in 2016, and (**c**) tanker links with low stability in 2016.

Figure 9 illustrates the spatial differentiation of links with evident flow dynamics (stability lower than 0.2) in the bulk, container, and tanker maritime network. As indicated in Figure 9a–c, there are some links that have high bulk flow dynamics between other countries (i.e., Ghana-United States, Panama-Colombia, among others) across different continents in 2016. The majority of the links around the Strait of Malacca presented high dynamics in the bulk maritime network, including the links among CN, SG, ID, and MY in 2014, AU, CN, SG, ID, and MY in 2015, as well as KR, AU, CN, SG, ID, and MY in 2016. In comparison with bulk links, the container links with high dynamics between other countries across Africa and South America and between 21C-MSR countries around the Strait of Malacca both decreased as illustrated Figure 9b. As illustrated in Figure 9c, the links between other countries around the English Channel, the straits in Turkey, Gulf of Mexico, and Panama Canal, as well as the links between 21C-MSR around the Strait of Gibraltar and the Strait of Malacca appeared to have high dynamics in tanker maritime network in 2016. This spatial variation might be driven by the different supply-demand structure and trends for different types of cargo transportation among multiple routes. Additionally, there are continuously increasing links with low stability in all of bulk, container, and tanker layers of 21C-MSR maritime network between 2014 and 2016, while other countries show an opposite trend.

#### 4.2.2. Spatial-temporal Dynamics of Maritime Network Structure

**Figure 10:**
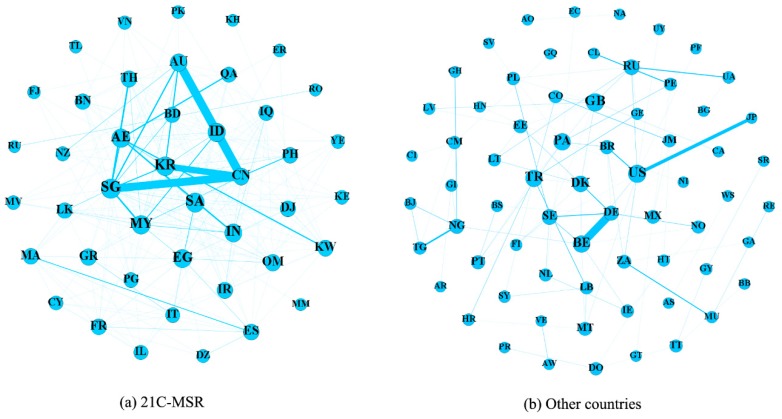
Maritime network structure variations; (**a**) 21C-MSR and (**b**) other countries.

Spatial-temporal dynamics of maritime network structures of 21C-MSR and other countries are summarized in Figure 10. The nodes represent different countries with different degree in the maritime network, and the widths of the links are characterized through their increasing accumulative weights derived from total capacity after normalization (divided by the maximum capacity). There are some differences in the overall evolutionary patterns of 21C-MSR in the time periods from 2014 to 2016. For example, China (CN) enhances the connection with Australia (AU), Malaysia (MY), and Indonesia (ID), and United Arab Emirates (AE) strengthens the interaction with India (IN), Kuwait (KW), Qatar (QA), Saudi Arabic (SA), and Singapore (SG). This indicates some countries in 21C-MSR enhanced the shipping connection between other countries in 21C-MSR between 2014 and 2016. Although there are corresponding countries continuously included by 21C-MSR, there are still many weak connections between 21C-MSR. This indicates it will still take a long tie for 21C-MSR to enhance mutual cooperation in their maritime shipping industry. The highest shipping connection between other countries is relatively low in comparison to 21C-MSR, and the highest increasing accumulative weights is also lower than 21C-MSR, as indicated figure 10. The maritime network structure of other countries consist of two centralities, one for United States (US) connected with Canada (CA), Colombia (CO), Mexico (MX), and Panama (PA), and another for European countries (e.g., United Kingdom (GB), Germany (DE), the Netherlands (NL), Belgium (BE)). There are no significant changes for shipping connections among other countries between 2014 and 2016. 

**Figure 11:**
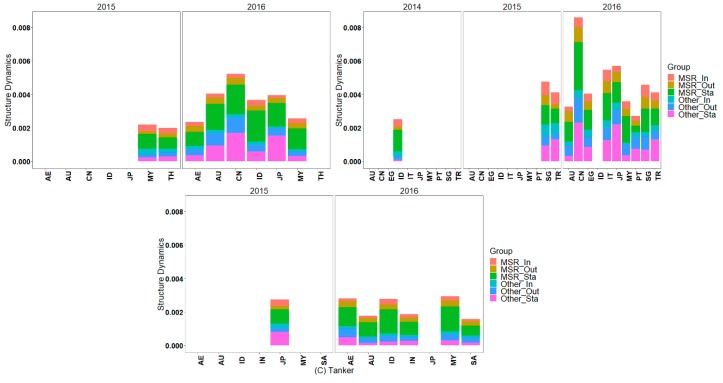
Maritime network structure with evident dynamics; (**a**) Bulk network, (**b**) container network, and (**c**) tanker network.

The maritime network can be decomposed into bulk, container, and tanker layers. The analysis will focus on the countries with network structure dynamics located in the top 20%. These countries carry out new business with additional ports (nodes) in 21C-MSR, and these new nodes contribute to the larger dynamics of the maritime network structure than the new nodes of other countries. However, these countries appear to reduce business with fewer ports in 21C-MSR, as missing nodes contribute to smaller dynamics of the maritime network structure than the missing nodes with other countries. This indicates that these countries exhibit evident dynamics in maritime network structures, especially for the shipping structure with 21C-MSR, which may be related to supply-demand shipping structure adjustment and carrying out new business with additional ports in 21C-MSR. It is obvious that there are more countries with a higher dynamic bulk, container, and tanker shipping network in 2016 than in 2014 and 2015. This is maybe related to the fact that the 21st-century Maritime Silk Road is still under construction and in the initial stages in 2014 and 2015, and the effectiveness of MSRI have been gradually presented since 2016. The maritime network structures with obvious dynamics include the bulk, container, and tanker of AU, MY, ID, and Japan (JP), bulk and container of CN, bulk and tanker of AE, bulk of TH, container of IT, PT, SG, and TR, and tanker of SA, as shown in Figure 11 (Note that “MSR_Sta” and “Other_Sta” represent the maritime network dynamics derived from the stable nodes with 21C-MSR countries and other countries, respectively; “MSR_In” and “Other_In” represent the maritime network dynamics derived from the new nodes with 21C-MSR countries and other countries, respectively; moreover, “MSR_Out” and “Other_Out” represent the maritime network dynamics derived from the missing nodes with 21C-MSR countries and other countries, respectively). In addition, JP presents high dynamics in all layers of maritime network structure although it wasn’t included by 21C-MSR. Japan has held a skeptical attitude toward MSRI, and developed some policies and measures to maintain competitiveness in the international shipping industry.

#### 4.2.3. Spatial-temporal Dynamics of Traffic Flow Weighted Maritime Network Structure

**Figure 12:**
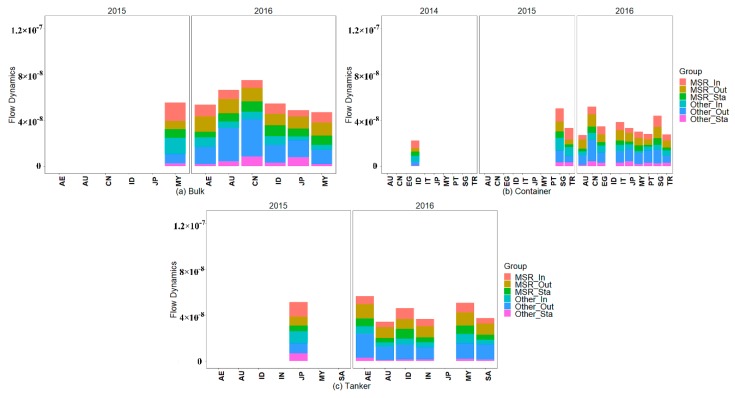
Traffic flow weighted maritime network structure with evident dynamics. (**a**) Bulk flow weighted maritime network, (**b**) container flow weighted maritime, and (**c**) tanker flow weighted maritime network.

The countries that have traffic flow weighted maritime network structure dynamics that ranked in the top 20% are illustrated in Figure 12. Additionally, these countries carry out new business with additional ports (nodes) in 21C-MSR, and these new nodes contribute larger dynamics of the traffic flow weighted maritime network structure than the new nodes with other countries. However, these countries close business with fewer ports in 21C-MSR, and these missing nodes contribute smaller dynamics of the traffic flow weighted maritime network structure than the missing nodes with other countries. This indicates their traffic flow weighted maritime network structures exhibit evident dynamics, especially for the shipping structure and capacity with 21C-MSR, which may be correlated with carrying out additional business with 21C-MSR. The traffic flow weighted maritime network structures emerging obvious dynamics include the bulk, container, and tanker of AU, MY, ID, and JP, bulk and container of CN, bulk and tanker of AE, container of EG, IT, PT, SG, and TR, and tanker of SA.

The bulk and container of CN, bulk container, and tanker of AU, MY, ID, and JP, bulk and tanker of AE, container of IT, PT, SG, and TR, and tanker of SA all exhibit evident dynamics in both the maritime network structure and traffic flow weighted maritime network structure, as illustrated in Figure 11 and Figure 12. This suggests that these countries built corresponding new shipping relationships with the ports in 21C-MSR, and these new linkages carried a significant amount of traffic flow between 2013 and 2016. The bulk of TH exhibit evident dynamics in the maritime network structure, but small dynamics in the traffic flow weighted maritime network structure. Therefore, TH built new bulk shipping linkages with numerous ports in 21C-MSR, but these new linkages carry only a small part of the traffic flow. The container of EG exhibits small dynamics in the maritime network structure but evident dynamics in the traffic flow weighted maritime network structure, which indicates that EG builds new container relationships with certain ports in 21C-MSR, and these new linkages carry significant traffic flow.

## 5. Conclusions

Understanding multi-layer maritime network dynamics is an initial step to predict change trend [1]. In this study, we have proposed a spatial-temporal framework to explore multi-layer maritime network dynamics, implemented the proposed framework using complex network theory and traffic flow stability, and investigated the spatial-temporal dynamics of nodes, links, network structure, and traffic flow between 2013 and 2016. The results are as follows. First, there are more ports in 21C-MSR countries that have high bulk, container, and tanker capacity and high changes in bulk, container, and tanker traffic flow between 2013 and 2016. This indicates in some ports in 21C-MSR countries, there exists a high shipping dynamic between 2013 and 2016. Additionally, most of the countries have a higher ports average capacity and stability after being included in 21C-MSR, which may be related to more frequent interaction between ports inside 21C-MSR. Second, there are more links with continuously increasing transport amount in all layers of maritime network in 21C-MSR countries compared to other countries, and these bulk, container, and tanker links in 21C-MSR countries present a higher average increased capacity compared to other counties. This illustrates that some of the linkages between 21C-MSR countries present an incremental shipping capacity between 2013 and 2016. Third, there are more links that have a big flow dynamics in bulk, container, and tanker maritime networks between 21C-MSR countries than between other countries. This indicates there are more links under the 21C-MSR geographic scope existing flow variation between 2013 and 2016. Fourth, the global maritime network dynamics exhibit geographical and spatial variations. For example, there are fewer container trade linkages with high dynamics between 21C-MSR countries around the Strait of Malacca than bulk linkages. Finally, certain countries (CN, SG, AU, and AE) have established new corresponding shipping relationships with some ports in 21C-MSR, and these new linkages carry substantial traffic flow between 2013 and 2016.

Although this research is investigating the spatiotemporal changes of the maritime network, extension may be possible. Geographical heuristics, place, and ship interaction dynamics in maritime transportation management and planning may be informative as would accounting for national shipping transportation strategies taking geopolitics into consideration. This research nevertheless provides policy insights. First, incremental transport amount of some ports and links in 21C-MSR countries between 2013 and 2016 may be relative to the shipping strategy adjustment. However, it is still premature whether 21C-MSR countries will become more competitive than other countries, and possibly hold a better position in the maritime trade. This indicates that the maritime transportation infrastructure, operational efficiency, and shipping routes for 21C-MSR countries can be further improved. The enhanced capacity for some ports maybe have potential effects for the nearby ports due to competitiveness, thus, maritime shipping policy development will need to account for the possibility of benefits conflicts among some ports in 21C-MSR countries. Second, maritime network dynamics are very useful for guiding global maritime shipping network improvements towards better utilization, including reducing friction in maritime trade and network shockwave both in 21C-MSR and other countries. Third, global maritime network dynamics provide some guidance for policy makers and stakeholders in decisions making as complicated maritime transportation markets reflect important structure and traffic flow evolution. For example, the ports or links with incremental transport capacity will cause transit time changes for shipping companies, thus, the adjustment is needed to maximize the benefits.

There are some limitations that should be noted. First, we only examine the global maritime network dynamics between 2013 and 2016, the difference between 21C-MSR and other countries, and the differences between before and after included by 21C-MSR across study period. The differences between before and after the MSRI was announced can be further explored if the long run AIS data are available. The spatial-temporal dynamics can be further evaluated if the complete data source is accessible. Second, this paper only focused on the multi-layer maritime network changes, and some uncertainty and challenge remains regarding implications for the actual impact of the MSRI on the maritime network. Third, there are some differences between shipping capacity and actual cargo amount owing to unknown cargo amount and loading rate. Fourth, this paper could not access the detail classification of goods and fixed importing and exporting countries for different products (e.g., crude oil and refined oil), which can be fulfilled in the future research. 

In the future, the proposed approach could be enhanced by combining comprehensive information on maritime natural resource utilization data, social-cultural factors, and economic activities in order to provide a powerful and mutually consistent explanation for the manner in which geopolitical initiatives have different impacts on maritime shipping planning and management [39]. Furthermore, future research should explore a deeper understanding of the mechanisms driving the structural and spatial and regional dynamics in global maritime networks, analyzing the urban transportation contributed by the geopolitical policy, and connecting these changes to the corresponding maritime network types.

## Figures and Tables

**Table 1:** Summary of research on maritime network structure.

Reference	Focus	Network Types	Indicators	Other Methods	Area
Li et al. [12]; Ducruet and Notteboom [13]; Xu et al. [14]	Structure and evolution	Container	Number of nodes, path length, mean journeys; degree, centrality, weighted centrality, clustering coefficient, eccentricity, rich-club coefficient, modularity, beta index, gamma index, gini coefficient, comprehensive centrality		World
Laxe et al. [15]	Structure and evolution	Container;			Sample of world fleet
Liu et al. [18]; Woolley-Meza et al. [19]; Lhomme [20]	Structure and spatial heterogeneity; Structure and robustness	Singer layer			World
Ducruet [2]	Structure and dynamics	Multilayer			World
Zhao et al. [32]; Caschili et al. [33]	Structure	Container;			Sample of world fleet
Ducruet [24]	Structure and diversity	Multilayer			world
Kaluza et al. [23]	Structure	Multilayer		Gravity model	Sample of world fleet
Tsiotas and Polyzos [34]	Structure and node aggregation	Tourism			Greece
Ducruet et al. [35]	Structure	container			East Asia
Kosowska-Stamirowska et al. [16]	Structure and evolution	Trade		Random walk	world
Liu et al. [21]; Calatayud et al. [22]	Structure and robustness	Container		Borda count	Maersk shipping line; Americas
Peng et al. [25]	Structure and robustness	Multilayer			world
Peng et al. [17]	Structure and evolution	Crude oil			world
Yu et al. [11]	Structure	Trade	Linkage intensity, linkage tightness, spatial isolation index, linkage concentration index		China

**Table 2:** Data categories in OD dataset of vessels.

Item	Meaning
Maritime Mobile Service Identity (MMSI)	Unique ID for the vessel
Start Time (Ship entering the port)/End Time (Ship leaving the port)	Second-level timestamp (e.g., 2015-06-10 01:16:58)
ship’s Location	Longitude and latitude of the ship location
Vessel_type	Type of vessel (bulk/container/tanker)
Vessel_name	Name of the vessel
Grosstone	Gross tonnage of the vessel
Length	Length of the vessel
Width	Width of the vessel
Draft	Draft of the vessel
Deadweight	Dead Weight of the vessel

**Table 3:** Links with continuously increasing capacity.

Type	Number	Average Increased Capacity	Main Countries
Bulk links with continuously increasing capacity in 21C-MSR.	345	3,125,407.29	AU, CN, ID, KR, SG
Bulk links with continuously increasing capacity in other countries.	236	1,243,252.48	Canada (CA), Ukraine(UA), United States (US)
Tanker links with continuously increasing capacity in 21C-MSR.	448	1,696,798.74	AE,CN, KR, KW, SG
Tanker links with continuously increasing capacity in other countries	450	1,408,144.76	Belgium(BE), Denmark(DK), United Kingdom(GB), the Netherlands (NL), Panama(PA), Sweden (SE), US

**Table 4:** Links with continuously increasing container capacity.

Type	Number	Average Increased Capacity	Main Countries
Container links with continuously increasing volume in 21C-MSR	388	3,524,387.11	CN, KR, MY, SG, TH
Container links with continuously increasing volume in other countries	250	2,320,540.44	CA,GB, NL, PA
